# A critical review on arsenic removal from water using iron-based adsorbents

**DOI:** 10.1039/c8ra08512a

**Published:** 2018-11-27

**Authors:** Linlin Hao, Mengzhu Liu, Nannan Wang, Guiju Li

**Affiliations:** College of Marine and Environmental Sciences, Tianjin University of Science & Technology Tianjin 300457 P. R. China liguij@tust.edu.cn; Department of Chemistry, National University of Singapore 3 Science Drive 3 Singapore 117543; School of Mechanical Engineering, Beijing Institute of Petrochemical Technology, Beijing Key Laboratory of Pipeline Critical Technology and Equipment for Deepwater Oil & Gas Development Beijing 102617 P.R. China

## Abstract

Intensive research efforts have been pursued to remove arsenic (As) contamination from water with an intention to provide potable water to millions of people living in different countries. Recent studies have revealed that iron-based adsorbents, which are non-toxic, low cost, and easily accessible in large quantities, offer promising results for arsenic removal from water. This review is focused on the removal of arsenic from water using iron-based materials such as iron-based nanoparticles, iron-based layered double hydroxides (LDHs), zero-valent iron (ZVI), iron-doped activated carbon, iron-doped polymer/biomass materials, iron-doped inorganic minerals, and iron-containing combined metal oxides. This review also discusses readily available low-cost adsorbents such as natural cellulose materials, bio-wastes, and soils enriched with iron. Details on mathematical models dealing with adsorption, including thermodynamics, kinetics, and mass transfer process, are also discussed. For elucidating the adsorption mechanisms of specific adsorption of arsenic on the iron-based adsorbent, X-ray photoelectron spectroscopy (XPS) and X-ray absorption spectroscopy (XAS) are frequently used. Overall, iron-based adsorbents offer significant potential towards developing adsorbents for arsenic removal from water.

## Abstract 2

The recent developments on iron-based adsorbents such as iron oxyhydroxides nanoparticles, zero-valent iron, bimetallic oxides, and iron oxyhydroxide-doped composite materials are fully discussed in this review.

## Graphical abstract

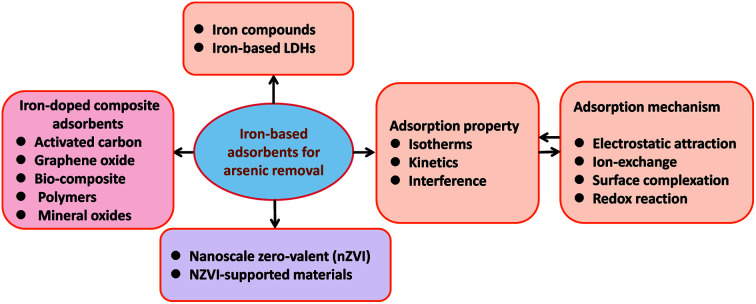

## 1. Introduction

### 1.1. Arsenic element in Nature

Arsenic element exists as oxides in the soil, sediments and water in many parts of the world and originates from both natural and anthropogenic activities. There are four chemical oxidation states for arsenic (−3, +3, 0, and +5) in Nature.^1^ The most common arsenic compounds that naturally occur are arsenite (H_3_AsO_3_ – As(iii)) and arsenate (HAsO_4_^2−^ – As(v)). As(v) is the predominant species present under oxidizing conditions and exists as oxyanions of arsenic acid (H_3_AsO_4_, H_2_AsO_4_^−^, HAsO_4_^2−^and AsO_4_^3−^), while As(iii) exists as arsenious acid (H_3_AsO_3_, H_2_AsO_3_^−^, HAsO_3_^2−^) under mildly reducing conditions.^2^ Arsenic compounds have been recognized as group 1 carcinogens by the International Agency for Research on Cancer (IARC).^3^ The current standard for the maximum contaminant level (MCL) of arsenic in drinking water recommended by the World Health Organization (WHO) is 10 μg L^−1^.

Arsenic species are always pH dependent.^4^ As(iii) exists mostly as neutral H_3_AsO_3_ when the solution pH is lower than 9.2 (the p*K*_a1_ of H_3_AsO_3_ is 9.2), while the dominant species of As(v) are H_2_AsO_4_^−^ and HAsO_4_^2−^ (the p*K*_a1_ of H_3_AsO_4_ is 2.3; the p*K*_a2_ of H_3_AsO_4_ is 6.8; the p*K*_a3_ of H_3_AsO_4_ is 11.6). As(iii) is about 60 times more toxic than As(v),^5^ and the mobility of As(iii) is more than that of As(v) because the probability of adsorption of neutral As(iii) to a mineral surface is less than As(v).^6^ Therefore, chemical oxidants such as chlorine, hydrogen peroxide (H_2_O_2_), ozone (O_3_), permanganate, and persulfate-based systems were frequently employed to oxidize As(iii) to As(v).^7^ For example, Zhou *et al.* (2017)^8^ and Hussain *et al.* (2017)^9^ has identified that the capacity of sodium persulfate (PS), activated by zero-valent iron (ZVI) to remove arsenic from water is much greater than that of PS alone due to the production of sulfate radicals. L. Zhou *et al.* (2013)^10^ also demonstrated that Fe(ii)/persulfate oxidation could be an effective method to oxidize As(iii) for the remediation of arsenic contaminated groundwater.

**Fig. 1:**
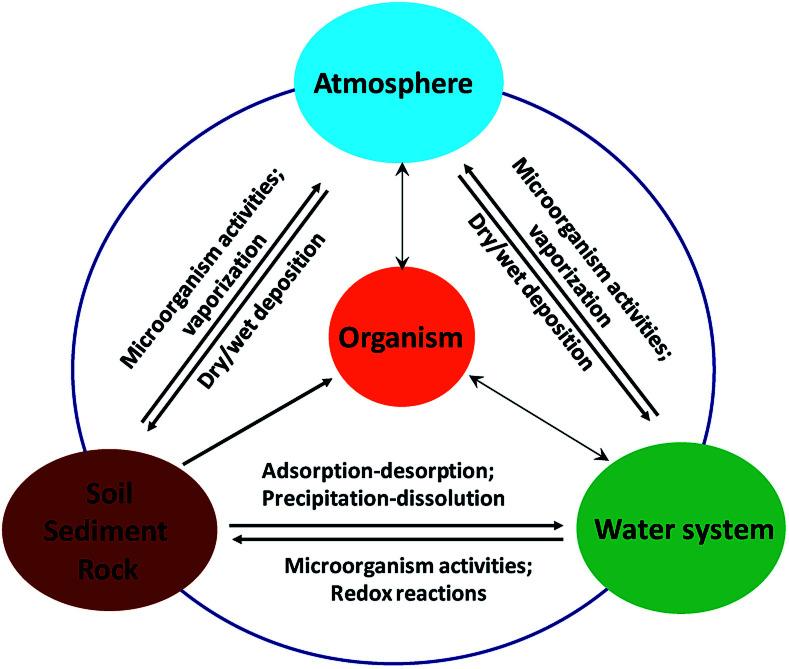
The geochemical cycles of arsenic in Nature.

Arsenic can release into the aquatic environments by natural processes such as dissolution of minerals by weathering, microbial activity, and complexation with natural organic materials.^11^ On the other hand, anthropogenic activities, including industrial mining and metallurgical industries, combustion of fossil fuels, use of arsenic pesticides, herbicides, and crop desiccants, can result in arsenic contamination in soils and surface water.^12^ The microorganism plays an important role in transformation of minerals or weathering of rocks in the geo-aqueous solution. Furthermore, it is reported that biogeochemical activities of microorganism can control arsenic contamination in groundwater by forming arsenical biominerals, such as loellingite (FeAs_2_) and symplesite (Fe_3_(AsO_4_)_2_·8H_2_O).^13^ Spratlen *et al.*^14^ and Oremland and Stolz^15^ proposed that even though arsenic is highly poisonous, certain prokaryotes use arsenic oxidation for energy generation either by oxidizing arsenite or by respiring arsenate. However there is limited research on arsenic mineral dissolution with considering both of chemical and microbial process in natural environment. Arsenic circulation in Nature is schematically shown in Fig. 1.

### 1.2. The distribution of arsenic in natural waters

#### 1.2.1. Groundwater

Arsenic pollution in groundwater at elevated concentrations is well documented in many countries such as America, Argentina, Bangladesh, Chile, China, India and Mexico at a concentration range from 1 μg L^−1^ to 73.6 mg L^−1^.^16^ As shown in Fig. 1, arsenic enters into groundwater in the form of As(iii) and As(v) through many ways such as industrial activities, weathering of rocks, volcanic emissions, biological activities and geochemical reactions.^17^ Arsenic exists in the natural pH range of groundwater primarily as an oxyanion of H_3_AsO_3_ that is neutral in charge. Owing to the deficiency of potable water sources, the arsenic contaminated groundwater (including geothermal water) was used for developing or underdeveloped world without treatment, which led to many adverse health conditions in the local population.

#### 1.2.2. Surface waters

The baseline concentrations of arsenic in US rivers or lakes have been reported in the range of 0.1–2.0 μg L^−1^.^18^ Gomati river (Ganga Plain, northern India) had arsenic concentrations in the range of 1.29–9.62 μg L^−1^ due to the geothermal influence and anthropogenic causes.^19^ Arsenic levels of 0.97–3.6 μg L^−1^ were found in water from Zenne River (Belgium), which was contaminated by As-containing sewage.^20^ Alpine/Mediterranean Var River water (France) showed arsenic concentrations in the range of 0.1–263 μg L^−1^.^21^ In the Stampede and Slate Creek watersheds (USA, Alaska), arsenic concentrations in stream waters were as high as 239 μg L^−1^ in the year of 2010.^22^ Manchar Lake (Pakistan) was reported to have arsenic concentrations of around 60.45 μg L^−1^.^23^ Moreover, sudden water pollution accidents through anthropogenic sources occur frequently owing to the development of global economy and urbanization processes.^24^

### 1.3. Harmful effect of arsenic pollution

Drinking water is the main source of arsenic exposure to the living organisms. Continuous exposure to arsenic pollution has been shown to cause damage to the central nervous system, kidney, skin, liver and lungs in humans.^25^ Arsenic can also increase glutathione peroxidase and mitochondrial superoxide dismutase (MSOD) activities in liver and lungs. In addition, chronic arsenic toxicity can cause cardiovascular diseases, hypertension and affects vascular system.^26^ Long-term contact with arsenic contaminated water can lead to pigmentation of the skin and development of hard patches on the palm of humans.^27^ To mitigate this situation, World Health Organization has reduced the maximum contaminant limit (MCL) of arsenic in drinking water from 50 to 10 μg L^−1^.^28^ Therefore, the development of more effective water treatment is required to satisfy the new regulations.

### 1.4. Methodologies for arsenic removal from water

**Fig. 2:**
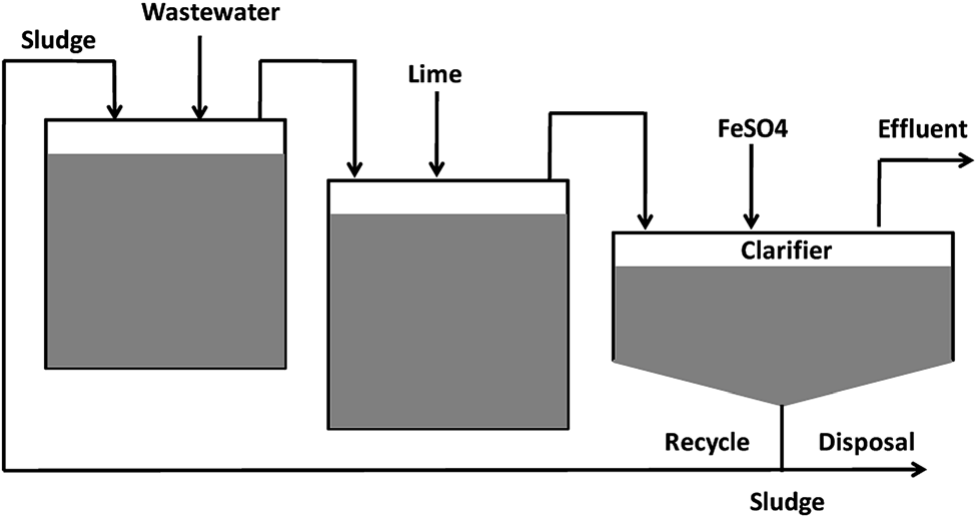
A typical method for the removal of high concentrations of arsenic from water.

Arsenic removal methods include chemical precipitation/flocculation,^29^ adsorption,^30^ ion exchange,^31^ reverse osmosis^32^ and electro-dialysis.^33^ Arsenic can be removed by precipitation as ferric arsenate, calcium arsenate or arsenic sulfide. It was established that As precipitation with ferric salts is more efficient than aluminium salts.^34^ However, the concentration of arsenic below 10 μg L^−1^ is usually difficult to attain *via* chemical precipitation. Moreover, removal of As(iii) during precipitation is considerably less effective than As(v) anions under similar conditions, and pre-oxidation is required to convert As(iii) to As(v) ions in water.^35^ For water containing high arsenic concentrations, lime softening was an effective way to lower the arsenic concentration, followed by a use of other techniques.^36^ The typical techniques for the removal of high concentrations of arsenic from wastewater are shown in Fig. 2. Klerk *et al.* (2015)^37^ conducted a continuous circuit co-precipitation of As(v) with ferric ions by lime neutralization. Two-stage continuous experiments (operating at pH 4 and 8, respectively) produced the lowest residual arsenic concentration when Fe/As molar ratio was kept at 4.

Ion exchange technology was also considered as another effective method to remove arsenic from water by using anion exchange resins.^38^ However, it is only efficient for As(v) removal, not good for the uncharged As(iii) species in water. Moreover, developing ion exchange resin and the high-tech water purification systems are usually expensive. The adsorption capacity was limited because of the interference from competitive adsorption of other co-existing anions. The adsorbent regeneration process also created a sludge disposal problem.

In recent years, membrane techniques, including nanofiltration and reverse osmosis, are increasingly reported for arsenic removal from water.^39^ Such techniques have advantages of high-removal efficiency, easy operation and minimum toxic sludge generated during the process.^40^ But the initial investment and running cost are relatively high; in addition, high pressure is usually needed to force the contaminated water through the membranes. Moreover, the discharge of the concentrate, membrane fouling and flux decline are usually inevitable in the membrane process.^41^ The electro-dialysis was capable of removing both arsenic and other contaminants, but large amounts of insoluble coagulants were also deposited on the cathode.^42^

Among many techniques currently available for arsenic removal from water, the adsorption process is considered one of the most promising techniques because of low cost, high efficiency, and ease of operation.^43^ Iron-based adsorbents have been extensively developed and showed good removal efficiency for arsenic species from water.^44^ Some adsorbents such as granular ferric hydroxide (GFH) and zero-valent iron have been produced on an industrial scale as commercial adsorbents.^45^ However, most of the reported adsorbents seldom make the practical field applications despite their proven high efficiency of arsenic removal, owing to the interfering ions present in the water. For the iron-based adsorbents, the common anions such as Cl^−^, NO_3_^−^, SO_4_^2−^, CO_3_^2−^ were not observed to have a significant influence on arsenic adsorption due to the specific chemical reaction between arsenic and iron.^46^ It was reported that phosphate can strongly compete with arsenic for the adsorption sites, thus decreasing the arsenic adsorption capacity.^47^ The presence of organic matter, such as humic acid and fulvic acid, also showed negative effects on arsenic adsorption in terms of delaying the adsorption equilibrium.^48^

## 2. Development of iron-based adsorbents for arsenic removal

Iron-based adsorbents attracted interest owing to their high efficiency in arsenic remediation, environmental friendliness and abundance on earth. In this review, attention is given towards exploring new iron-based adsorbents with high adsorption capacities for As species and a summary of the relevant mechanism of adsorption. We have referred most of the valuable published literature on arsenic remediation by adsorption. Arsenic adsorption using iron compounds, zero-valent iron, iron-based bimetal oxides, iron-doped composite adsorbents are critically reviewed and their adsorption efficiencies are compared. Besides the adsorption capacities, the characteristics of adsorption processes, including thermodynamics, kinetics and mass transfer mechanisms are also examined. The specific binding between arsenic and iron was deeply investigated by various spectral technologies. Moreover, some iron-based adsorbents are magnetic, allowing for an easy separation of the saturated materials from water in an external magnetic field.

### 2.1. Iron oxy-hydroxides

**Fig. 3:**
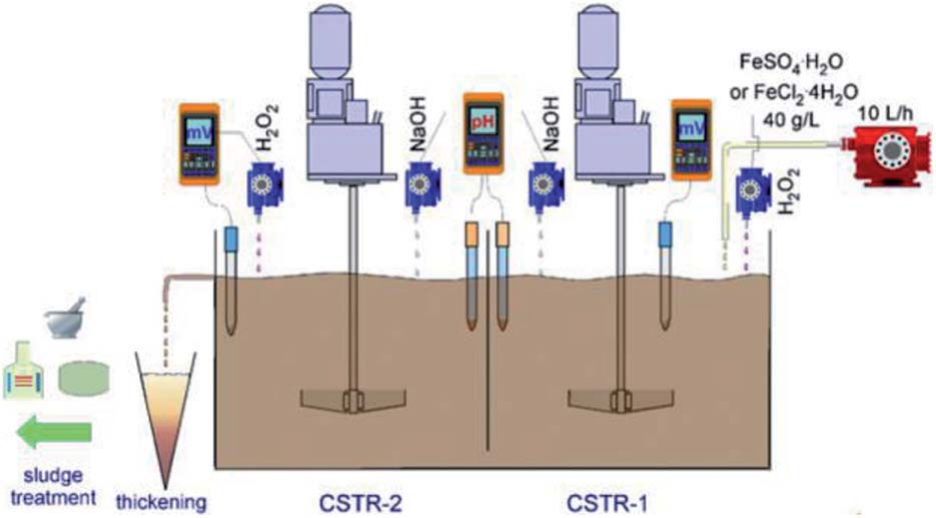
Scheme of the laboratory iron oxy-hydroxides' production.^52^

Many different materials have been reported to have a good affinity towards arsenic, but iron oxy-hydroxides are the most widely studied because of their easy accessibility. The commonly used iron oxy-hydroxides such as, akaganèite (β-FeOOH), goethite (α-FeOOH), lepidocrocite (γ-FeOOH), ferrihydrites (Fe_10_O_14_(OH)_2_), green rusts can be chemically synthesized by the precipitation of Fe(iii) or Fe(ii) salts through the hydrolysis and oxidation processes.^49–51^ A scale-up method for the preparation of iron oxy-hydroxide in large amounts with a high production yield using FeSO_4_·H_2_O and FeCl_2_·4H_2_O was reported.^52^ The synthetic route included a sequence of oxidation, hydrolysis and precipitation using a continuous flow reactor (as shown in Fig. 3), the synthesized adsorbents presented better performance for adsorption of As(iii) species as compared to commercial granular ferric hydroxide (GFH) and granular ferric hydroxide (GFO).^53^ Abiogenic iron oxy-hydroxide is reported to be more efficient to remove As(v) than biogenetic iron oxy-hydroxide.^54^ It is explained that nitrate-reducing Fe(ii)-oxidizing bacteria can use nitrate as an electron acceptor to oxidize Fe(ii) to precipitate the biogenic iron oxy-hydroxides in anoxic groundwater aquifers.

The photochemistry of As(iii) adsorption on ferrihydrite was investigated by using the attenuated total reflection Fourier transform infrared spectroscopy (ATR-FTIR) and X-ray absorption near edge structure (XANES).^55^ The stable As(iii) oxidation state in the dark is gradually transformed to As(v) on ferrihydrite in presence of light at pH 5. At the same time, Fe(iii) ions were reduced to Fe(ii) species during the As(iii) oxidation.^56^ It was observed the adsorption rate (12.4 × 10^−5^ M s^−1^ m^−2^) on goethite was significantly faster than that of ferrihydrite (6.73 × 10^−5^ M s^−1^ m^−2^) at pH 5.^57^ The similar photochemical reaction was also observed for As(iii) adsorption on goethite in the presence of dissolved oxygen.^58^ Laterite, a natural iron oxide mineral containing 91% of goethite, is another promising and cost-effective material for arsenic adsorption.^59^ The specific adsorption of arsenic is demonstrated by a strong evidence of the shift of isoelectric point. Iron-rich laterite was more effective than goethite (α-FeOOH), magnetite (Fe_3_O_4_) and hematite (Fe_2_O_3_), because of the higher specific surface area (81.2 m^2^ g^−1^).^60^ Natural siderite has been widely studied to remove both As(iii) and As(v) species from water,^61^ but the adsorption rate and capacity were relatively low. For example, arsenic adsorption on natural siderite with the particle size of 0.10–0.25 mm, reached equilibrium in 3 days, and the estimated maximum adsorption capacity was only 1.04 and 0.52 mg g^−1^ for As(iii) and As(v), respectively.^62^ However, As(iii) adsorption on the synthetic siderite is fast and the adsorption equilibrium can be reached in 20 min.^63^ As the percentage of oxidized As(iii) increased, the siderite was converted to lepidocrocite and goethite. Moreover, when the natural siderite was modified with polyanionic cellulose, the adsorption capacity and adsorption rate can be greatly increased.^64^

In most of the cases, arsenic adsorption on iron compounds could fit the Langmuir model better than Freundlich model.^65^ For example, As(iii) adsorption on hematite,^66^ As(iii) and As(v) adsorption on magnetite,^67^ goethite,^68^ amorphous iron hydroxide^69^ and magnetite–maghemite nanoparticles^70^ could be well described by Langmuir model, indicating the monolayer adsorption on energetically equivalent sites. However, As(v) adsorption on granular ferric hydroxide (GFH) fitted Freundlich model better with a high correlation coefficient (*R*^2^ > 0.99),^71^ indicating the heterogeneous active sites distributed on GFH.

**Fig. 4:**
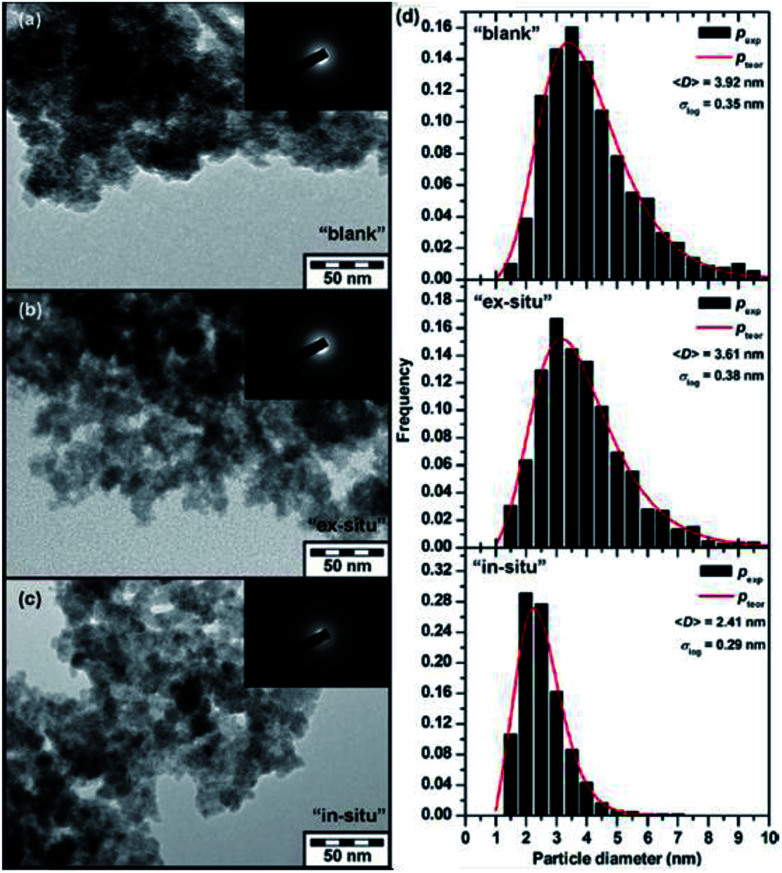
TEM images of the (a) “blank”, (b) “*ex situ*”, and (c) “*in situ*” samples and (d) particle size distribution of all three studied systems derived from the statistical processing of TEM images. Note: “Blank” sample formed after addition of ferrate(vi) only to deionized water, “*in situ*” sample formed after simultaneous addition of ferrate(vi) and an As(v)-containing compound to deionized water, and (iii) “*ex situ*” sample formed after addition of ferrate(vi) to deionized water and followed by an addition of As(v)-containing compound.^83^

The dissolved O_2_ and Fe(ii) ions have a significant impact on the adsorption of As(iii) and As(v) species on lepidocrocite (γ-FeOOH).^72^ Lepidocrocite can release Fe(iii) ions into the water during the adsorption and oxidation of As(iii) species. It was reported the adsorbed As can be incorporated into the lattice of γ-Fe_2_O_3_ nanoparticles,^73^ the in-field ^57^Fe MÖssbauer spectra and TEM results confirmed that the incorporated As(v) ions inhibited the nanoparticle growth resulting in a low average size of the formed γ-Fe_2_O_3_ nanoparticles (as shown in Fig. 4). The δ-FeOOH with a surface area of 135 m^2^ g^−1^ exhibited an As(v) adsorption capacity of 37.3 mg g^−1^ at a pH 7.0.^74^ The kinetics data were best fitted with a pseudo-second-order, thus suggesting the formation of inner-sphere complexes between As(v) and δ-FeOOH nanoparticles.

Among the polymorphs of FeOOH such as goethite (α-FeOOH), lepidocrocite (γ-FeOOH) and akaganèite (β-FeOOH), akaganèite showed the highest adsorption capacity for arsenic.^75^ Akaganèite with a surface area of 330 m^2^ g^−1^ showed adsorption capacity as high as 120 mg g^−1^ at pH 7.5.^76^ The main composition of commercial granular ferric hydroxide (GFH) is β-FeOOH. Table 1 shows the adsorption capacities of arsenic on different iron compounds.

**Table 1:** The list of different iron compounds used for arsenic adsorption from water

Adsorbents	Surface area (m^2^ g^−1^)	Initial conc. (mg L^−1^)	pH	Adsorption capacity (mg g^−1^)		Ref.
				As(iii)	As(v)	
Granular ferric hydroxide (GFH)	240–300	As(v): 0.1	6.5–7.5	—	1.1	53
α-FeOOH nanoparticles	167.8	As(v): 100	3.0	—	76	68
Ultrafine α-Fe_2_O_3_ nanoparticles	162	—		47	95	73
Ultrafine δ-FeOOH	135	As(v): 20	7.0	—	37.3	74
β-FeOOH nanoparticles	330	As(v): 20	7.5	—	120	76
Magnetite–maghemite nanoparticles	49	As(iii): 1.5	2.0	3.69	3.71	77
		As(v): 1.5				
α-Fe_2_O_3_		As(v): 1	3–10	—	0.2	78
Fe_3_O_4_ nanoparticles	179	As(iii): 70	5.0	16.56	46.06	79
		As(v): 25				
γ-Fe_2_O_3_ nanoparticles	41–49	As(v): 1	7.0	—	2.9	80
Fe_3_O_4_–γ-Fe_2_O_3_ nanoparticles	60	As(iii): 1.5	2.0	3.69	3.71	81
		As(v): 1.5				

### 2.2. Iron-based layered double hydroxides (LDHs) as adsorbents

Iron-based LDHs, incorporating other metals, such as Mg, Ni, Zn, Mn and Co ions into iron oxides, have gained much more interest for arsenic adsorption due to the synergistic effect and the considerable higher adsorption capacities.^82^ The chemical compositions of their layer cations and their interlayer anions can be greatly varied, and the interlayer space can be explored for arsenic removal from water.^83^ The general formula of iron-based bimetal oxides is:
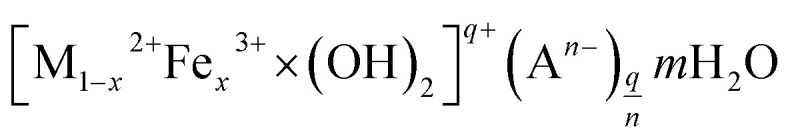
where M^2+^ represents metallic divalent cations such as Mg(ii), Co(ii), Zn(ii) and Mn(ii); A^*n*−^ is the interlayer anion of charge *n*.

The most popular method of LDHs preparation is the direct co-precipitation, which is based on hydrolysis of two metal cations (*e.g.*, Mg and Fe) by strong bases in the presence of another precursor that contains potential interlayer anions, such as carbonate (CO_3_^2−^).^84^ Moreover, the combination of co-precipitation with other treatments such as ultrasound- and sono-assistances was frequently used to enhance adsorptive properties. The solgel method has been proven to be an effective strategy to produce high-quality LDHs.^85^ A nanostructured Fe–Ni-LDHs with a specific surface area of 245 m^2^ g^−1^ was synthesized as shown in eqn (1), using a co-precipitation/calcination techniques.^82^ The chemical transformation is given:1
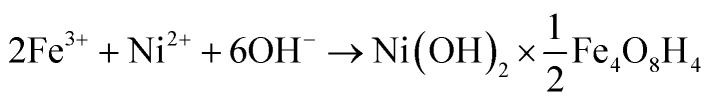


This Fe–Ni-LDHs exhibited very high adsorption capacity of 168.6 mg g^−1^ and 90.1 mg g^−1^ for As(iii) and As(v), respectively, which are higher than most of the reported iron-containing adsorbents. The addition of Ni contributed to the porous structure, high specific surface area and increased surface functional groups (such as Ni–OH, Fe–OH), thus greatly enhancing the arsenic adsorption efficiency.^86^ Micro-sized Fe–Cu-LDHs exhibited much higher arsenic adsorption capacity than those of the single iron oxide and copper oxide.^87^ Fe–Al-LDHs also showed higher arsenic adsorption capacity than single iron oxide.^88^ Fe–Mn-LDHs was effective for As removal from water because the positively charged manganese oxides surface attract and oxidize As(iii) to As(v) and allow increased adsorption of As(v) on iron oxides. A mesoporous Fe–Mn-LDHs was synthesized by using a hard template method.^89^ The maximum adsorption capacities of Fe–Mn-LDHs for As(iii) and As(v), calculated by Freundlich model, were 68 and 94 mg g^−1^, respectively. A porous nanobimetallic Fe–Mn cubes was synthesized by Zhang *et al.* (2017).^90^ The adsorbent showed a higher specific surface area of 450 m^2^ g^−1^ than that of 138 m^2^ g^−1^ reported by Zhang *et al.* (2010)^91^ and 197 m^2^ g^−1^ reported by Hu *et al.* (2017).^92^ The adsorption capacities of porous nanobimetallic Fe–Mn cubes for As(iii) calculated by Langmuir model, were 460 mg g^−1^. The removal mechanisms involved electrostatic attraction, surface complexation, and oxidation/adsorption due to the presence of MnO_2_ in the bimetal oxides. Lu *et al.* (2015)^93^ reported a Zn–Fe-LDHs to achieve the efficient removal of As(v) in aqueous solutions. The experimental result of simulated water samples showed that the adsorption of As(v) on Zn–Fe-LDH material can be well described by the Sips isotherm model with the maximum adsorption capacity of 151.37 mg g^−1^.

As(v) adsorption on mono-(Fe or Al) and Fe–Al-LDHs supported zeolite agreed with the Redlich–Peterson model with the correlation coefficient above 0.99.^94^ Similarly, As(iii) and As(v) adsorption on magnetic nanoscale Fe–Mn-LDHs loaded zeolite (MFM) could be well described by Redlich–Peterson model with the correlation coefficient above 0.98.^91^ When the atomic ratio of Mn/Fe was 2 : 9, the specific surface area of the Fe–Mn-LDHs nanoparticles reached 340 m^2^ g^−1^, which is higher than most of the reported absorbents and showed high As removal efficiency of 99.0% at pH 7.0.^95^ A list of arsenic adsorption on different iron-based LDHs are shown in Table 2.

**Table 2:** List of different LDHs and their arsenic adsorption capacities

LDHs	*S* ^BET^ (m^2^ g^−1^)	Atomic ratio	Adsorption capacity (mg g^−1^)		Ref.
			As(iii)	As(v)	
Fe–Ni	245	2 : 1	168.6	90.1	86
Fe–Cu	282	2 : 1	122.3	82.7	87
Fe–Al	87.4	1 : 1.7	40.6	37.6	88
Fe–Mn	154	3 : 1	68	94	89
Fe–Mn	450	3 : 1	460	—	90
Fe–Mn	340	9 : 2	342	—	95
Fe–Ce	90	3 : 0.8	—	150	96
Fe–Ce	265	3 : 1	72	133	97
Fe–Ti	77.8	4 : 1	65	15	98

### 2.3. Nanoscale zero-valent iron (nZVI) and nZVI supported adsorbents

In recent years, nZVI has become a hotspot of research in many fields due to its high reactivity with a standard redox potential of −0.44 V.^99^ NZVI can degrade the organic pollutants in presence of dissolved oxygen (DO) by transferring electrons to O_2_ to produce oxygen radicals (O_2_˙) and H_2_O_2_ in the medium. Moreover, the combination of H_2_O_2_ and Fe^2+^ can produce hydroxyl radicals (OH˙) which possess the strong oxidizing ability. The mechanism is shown as follows:^100^

2 Fe^0^ + O_2_ + 2H^+^ → Fe^2+^ + H_2_O_2_

3 Fe^0^ + H_2_O_2_ + 2H^+^ → Fe^2+^ + 2H_2_O

4 Fe^2+^ + H_2_O_2_ → Fe^3+^ + OH˙ + OH^−^

However, nZVI tends to agglomerate in solution, which causes a reduction in reactivity. Direct usage of nZVI in water also caused pollution because the nanoparticles themselves are considered as an emerging class of contaminants with a wide distribution in water system.^101^ Loading of nZVI onto appropriate supporting materials may reduce the leaching of nanoparticles into the water.

A bi-functional polystyrene resin supported nZVI was reported for the adsorption of As(iii) and As(v).^102^ The crosslinked polystyrene with a crosslinking density of 8% was used as matrix and the maximum adsorption capacities for As(iii) and As(v) reached 121 and 125 mg g^−1^, respectively. A nZVI-supported mesoporous carbon composite adsorbent was also reported recently and TEM images showed a homogeneous distribution of nZVI (10–20 nm) particles within the mesoporous carbon.^103^ The electron paramagnetic resonance (EPR) and proton binding measurements showed nZVI surface is partially oxidized to form an iron(iii) oxide/hydroxide shell, which was mainly responsible for As(iii) binding.^103^ A nZVI-supported montmorillonite was reported to show a maximum adsorption capacity of 59.9 and 45.5 mg g^−1^ for As(iii) and As(v), respectively.^104^ The co-existing anions, such as chloride and carbonate slightly decreased the removal of As(iii) to ∼90%, while nitrate and phosphate anions exhibited higher impact resulting in reduction of As(iii) removal efficiency to ∼80%.^105^

A nZVI impregnated chitosan-carboxymethyl β-cyclodextrin complex was also successfully tested for arsenic removal from water.^106^ NZVI nanoparticles were entrapped into chitosan-carboxymethyl β-cyclodextrin complex, which enhanced the stability of Fe^0^ particles and the carboxymethyl β-cyclodextrin provided more active sites to interact with arsenic species. The maximum adsorption capacity was calculated by Langmuir model and found to be 18.51 and 13.51 mg g^−1^ for As(iii) and As(v), respectively. Monodispersed nZVI particles could combine with chitosan fibres with an average fibre diameter of 195 ± 50 nm to produce functional and stable adsorbent.^107^ The nZVI doping on chitosan surface was typically achieved through a liquid phase reduction of FeCl_3_ using NaBH_4_. The reaction mechanism is shown as follows:

5 4Fe^3+^ + 3BH^−^_4_ + 9H_2_O → 4Fe^0^↓ + 3H_2_BO^−^_3_ + 12H^+^ + 6H_2_

The XPS analysis revealed that arsenic was fixed to oxy-hydroxide groups at the outer shells of nZVI surfaces, while As(iii) underwent oxidation to As(v). The environmental risk of As-loaded nZVI was evaluated by Ye *et al.*^108^ The results indicated that an aerobic As(v)-reducing bacterium (*Pantoea* sp. IMH) preferentially reduce soluble As(v), not solid-bound As(v). Nanoscale zero-valent iron was supported onto activated carbon (NZVI/AC) for arsenic removal from drinking water. The results showed that the iron particles in the pores of carbon were needle-shaped with the size of (30–500) × (1000–2000) nm. The maximum adsorption capacity for As(iii) and As(v) at pH 6.5 calculated from Langmuir model was 18.2 and 12.0 mg g^−1^, respectively.^109^ Wu *et al.*^110^ investigated the double influence mechanism of pH on arsenic removal by nZVI (with an average particle size varying from 30 to 50 nm). The results indicated that an increasing pH decreased the γ-FeOOH and increased the Fe_3_O_4_/γ-Fe_2_O_3_ content in the corrosion products of nZVI, thus enhancing the adsorption affinity of nZVI to As(v). The iron loading of fuller's earth immobilized nZVI (F-nZVI) were synthesized by borohydride reduction method,^111^ the maximum adsorption capacity of F-nZVI for As(iii) and As(v) were observed to be 50.1 and 90.4 mg g^−1^, respectively. The representative studies for arsenic removal by using nZVI or supported nZVI are listed in Table 3.

**Table 3:** Representative studies for arsenic removal by nZVI and supported nZVI

Adsorbent	pH	Initial	Adsorption capacity *q*_max_ (mg g^−1^)		Ref.
		Conc. (mg L^−1^)	As(iii)	As(v)	
N/S-nZVI	6.5	1–100	121	125	102
NZVI/AC	7	9	26.8	—	103
NZVI/mont-morillonite	7.0	5	59.9	45.5	104
NZVI	7.0	0.2	1.8–2.0	—	105
NZVI	7.0	—	3.5	—	108
NZVI/AC	6.5	2	18.2	12.0	109
NZVI	6.0	2–100	1.7	0.7	110
F-nZVI	7.2	100	50.1	90.4	111

### 2.4. Iron oxy-hydroxides doped composite adsorbents

Nanomaterials received substantial attention in the area of water treatment owing to the high surface area and interesting catalytic properties.^112^ Iron oxy-hydroxide nanoparticles are promising adsorbents for arsenic removal because of the high reactivity, and non-toxic nature.^113^ However, direct addition of these nanoparticles into water is not feasible owing to the difficulty in removing them from the water after adsorption process. A few nanoparticles such as engineered silver nanoparticles and graphene oxide (GO) are known to be toxic to the living systems.^114^ To overcome this problem, the supporting materials such as granular activated carbons, biomass materials, polymers, zeolite, silica, clay mineral, and red mud have been extensively used to combine with iron oxide nanoparticles for arsenic adsorption from water.^115^

#### 2.4.1. Iron oxy-hydroxides doped activated carbon

Granular activated carbon (GAC) is most frequently used to remove organic pollutants from water due to its high specific surface area.^116^ But GAC poorly adsorbs arsenic species because of its negatively charged surface.^117^ Iron modified activated carbons have been employed to enhance arsenic adsorption capacity in the past decades. In these composite adsorbents, iron oxide particles are the active components for arsenic removal whereas GAC provides a high surface area and acts as a solid support. Such combination of iron oxide nanoparticles and GAC was demonstrated to be a feasible method to take advantage of the properties of two materials for arsenic adsorption.^118^

In order to improve arsenic adsorption, GAC impregnation using a solution of iron salt is most frequently used to synthesize iron oxide doped GAC.^119^ Lee *et al.* (2015)^120^ reported iron oxides incorporated activated carbon for As(v) removal from water by hydrothermal method. It was indicated that the Redlich–Peterson model was the most suitable model for describing the equilibrium data. Experimental factors such as nature of iron salt, concentration, pH, and treatment time play key roles towards arsenic adsorption capacity. The adsorption mechanisms are associated with electrostatic attraction, ion exchange, and surface complexation.^121^ The arsenic adsorption was most efficient when the iron loading content on GAC was ∼6%, further increases in iron content unexpectedly decreased the arsenic adsorption capacity.^122^ Phosphates and silicate anions significantly decreased arsenate removal at pH > 8.5, while sulfate, chloride, and fluoride anions had minimal effects.^123^

Hematite and akaganèite loaded GAC was synthesized to remove As(v) and As(iii) from water.^124^ The surface area and pore volume slightly decreased after doping the GAC with hematite and akaganèite nanoparticles due to the obstruction of micropores, but As(v) adsorption capacity was significantly enhanced after modification. Iron oxide nanoparticles decorated GAC was prepared using microwave-assisted hydrothermal technique and tested for water purification.^125^ Iron oxide deposited on GAC was characterized as β-FeOOH after 3 min, and β-FeOOH was gradually transformed to α-Fe_2_O_3_ after 6 min of heating. The mechanism of the synthetic route is proposed as follows:

6 AC^−^ + Fe^3+^ → AC + Fe^2+^

7 2Fe^2+^ + MnO_2_(s) + 2H_2_O → 2FeOOH(s) + Mn^2+^ + 2H^+^

The GAC treated with a FeCl_3_ solution of lower concentration (*i.e.* 0.05 M) was more efficient for removing arsenic than those treated with higher concentration (*i.e.* 0.2 mol L^−1^) of the FeCl_3_ solution.^126^ The use of Fe(ii) is favourable for obtaining higher Fe content inside the iron-doped activated carbons. The surface oxidation of GAC by concentrated HNO_3_/H_2_SO_4_ or HNO_3_/KMnO_4_ could greatly increase the densities of carboxylic or other functional groups on the surface. The iron loading amount correlated well with the number of surface carboxy- and hydroxyl-functional groups.^127^ The effect of experimental conditions (*i.e.*, pre-oxidation, contact time, and iron concentration) on the distribution and morphology of iron oxy-hydroxide on GAC was also examined.^128^ The authors indicated that the contact time and iron concentrations have no significant effect on iron loading content, the use of KMnO_4_ yielded teeth-like iron oxyhydroxide nanoparticles, while the absence of KMnO_4_ pretreatment produced spherical nanoparticles.^129^Table 4 shows the comparison of arsenic adsorption on different iron modified activated carbon.

**Table 4:** List of iron modified activated carbon (AC) prepared by different groups for arsenic adsorption

AC type	*S* ^BET^ (m^2^ g^−1^)	*S* ^BET^ (after iron loading)	Iron loading content (mg g^−1^)	Iron phase	Adsorption capacity (mg g^−1^)		Ref.
					As(iii)	As(v)	
Bituminous based Filtrasorb 400	929	863	0.95%	HFO	—	2.45	120
Coconut shell	667	388	1.5%	HFO	—	1.25	121
Commercial NC-100	2100	—	4.56%	Fe(ii)	0.035 (initial total As conc. is 0.31 mg L^−1^)		122
Commercial NC-100	2100	1575	2.2%	HFO	0.035 (initial total As conc. is 0.31 mg L^−1^)		123
ACF cloth	1720		3.56%	Fe_3_O_4_	—	4.16	124
Lignite-based AC			11.4%	Amorphous FeOOH	—	0.26 (initial As conc. is 0.12 mg L^−1^)	125
Wood-based BAX-1500	2143	918	8.5%	Amorphous FeOOH		32.96	126
Starbon300	213	141	5.6%	nZVI	26.8	—	127
F400 AC	896		1.31%	Amorphous HFO	—	4.56	128
Straw activated carbon	723		11.7%	Amorphous HFO	51.3	33.8	129
Darco 20 × 50	650	—	4.22%	β-FeOOH, amorphous HFO	—	1.95 (initial As conc. is 40 mg L^−1^)	130
Sawdust-based AC	—	349	39%	Fe_3_O_4_	—	204 (initial As conc. is 40 mg L^−1^)	131

#### 2.4.2. Iron oxy-hydroxides doped graphene oxide (GO)

β-FeOOH@GO-COOH (carboxylic graphene oxide) nanocomposite was used for the removal of arsenic from contaminated water.^132^ GO was prepared by oxidation of graphite, NaOH and ClCH_2_COOH were mixed with GO solution under sonication to produce carboxylic GO, the product was dispersed into anhydrous ethanol, mixed with FeCl_3_ and stirred at room temperature for 24 h under nitrogen atmosphere.^132^ The XRD pattern demonstrated that the iron oxide deposited on GO-COOH is β-FeOOH with the characteristic peaks at around (2*θ*) 35.2°, 39.2°, and 55.9°. The adsorbent provided high adsorption capacities of 77.5 mg g^−1^ for As(iii) and 45.7 mg g^−1^ for As(v), respectively. Guo *et al.* (2015)^133^ synthesized a three-dimensional Fe_3_O_4_–graphene composite for exploring arsenic adsorption. The 3D graphene xerogel was mixed with polydopamine to strengthen the macroscopic architecture of 3D graphene, so as to enhance the loading of Fe_3_O_4_ nanoparticles. The composite adsorbent with the Fe_3_O_4_ loading of 6.1% was separated using a magnet. The synthesized adsorbent was capable of removing low concentrations of arsenic (0.05 mg L^−1^) from water.^133^ Three-dimensional iron oxide nanostructures@graphene-carbon nanotubes were prepared through a highly versatile and one-pot microwave route and used for arsenic removal.^134^ The high mesoporosity and open pore network of the graphene-CNT matrix facilitate fast molecular diffusion and promote the accessibility to iron oxide particles. The XPS and Raman spectroscopy indicated the iron particles exist in a mixed state of Fe_2_O_3_ and FeOOH. A superparamagnetic magnetite on graphene composite was synthesized *via* chemical reaction with a magnetite particle size of ∼10 nm.^135^ The separation of the composite material was completed in ∼10 s under the applied magnetic field of ∼20 mT. The observed As(iii) adsorption capacity of 13.1 mg g^−1^ was higher than that of As(v) (5.83 mg g^−1^), indicating the arsenic adsorption process is controlled by surface complexation.^135^ A magnetic graphene oxide (MGO) composite was prepared with Fe_3_O_4_ uniformly deposited on the GO surface by mixing FeCl_3_ and FeCl_2_ solutions and exposing to ammonia solution.^136^ The reaction is shown as follows:

8 Fe^2+^ + 2Fe^3+^ + 8OH^−^ → Fe_3_O_4_↓ + 4H_2_O

The thermodynamic results indicated the adsorption of As(v) on MGO is an endothermic process and the kinetic data were fitted with the pseudo-second-order model. At low pH values, the co-existing anions showed an inhibiting effect while an enhancing effect was observed on As(v) adsorption at high pH values.^136^

#### 2.4.3. Iron oxy-hydroxides doped biocomposite adsorbents

Recently, the development and application of biocomposite materials are becoming more attractive due to the low cost and eco-friendliness. Biomass materials, such as spent grain, onion skin, rice husks, bark and sawdust, maize cobs, wheat bran, and insoluble starch have been utilized for the removal of arsenic species from water.^137–139^

Biochar is another material that can be obtained from pyrolysis of agricultural waste.^140^ Because of its easy availability and low cost, biochar has been considered as an alternative adsorbent for polluted water treatment.^141^ Biochar loaded with iron oxy-hydroxides particles act as a good adsorbent for the removal of arsenic pollutants from water.^142^ In a recent study, a walnut based biochar loaded with α-FeOOH was formed through direct hydrolysis of an iron salt and showed good adsorptive performance for arsenic from water.^143^ The iron impregnated biochar showed much better adsorption ability with maximum adsorption capacity of 2.16 mg g^−1^ than the pristine biochar with no As adsorption capacity. Also, the authors proposed the chemisorptions mechanism based on the evidence of large shifts in the binding energy of Fe2p, As3d, O1s and C1s region after As adsorption. The results indicated a change in chemical speciation of As(v) ions getting reduced to As(iii) species, and Fe(ii) was oxidized to Fe(iii) during the adsorption process.^144^ Another Fe-loaded biochar was derived from sugar beet pulp (BP) agricultural residues.^145^ The authors found that GAC, preoxidized by hydrogen peroxide (H_2_O_2_) or potassium permanganate (KMnO_4_), could significantly increase the iron loading amount from 5% to 10–32%. Apricot stone was activated by treating it with phosphoric acid (H_3_PO_4_) and carbonized under nitrogen flow led to the formation of biochars, which was modified with iron oxyhydroxides to prepare the hybrid adsorbents.^146^ The comparison of Fe(ii) loaded GAC (GAC-Fe(ii)) and Fe(iii) loaded GAC (GAC-Fe(iii)) for As(v) adsorption revealed that GAC-Fe(iii) has a better adsorptive performance for arsenic extraction than GAC-Fe(ii) adsorbent. More interestingly, the authors indicated that As(v) adsorption on GAC-Fe(ii) is an endothermic process, while As(v) adsorption on GAC-Fe(iii) is an exothermic process according to the values of enthalpy change (Δ*H*^Θ^).^147^

Chitin and chitosan are the most abundant biopolymers in Nature. Chitosan is derived from chitin, which is the main constituent of the exoskeleton of crustaceans.^148^ Chitosan has a strong affinity towards ferric ions, which further uptake arsenic species from aqueous mediums. Chitosan complexed with Fe(iii) ions showed the highest As(v) adsorption efficiency as compared to Cu(ii), La(iii), Mo(vi) and Zr(iv) complexed chitosan. The As(v) ion adsorption capacity of self-supported Fe(iii)-chitosan membrane reached 109 mg g^−1^.^149^

Yamani *et al.* (2014)^150^ reported a Fe_3_O_4_@Zr(OH)_4_ impregnated chitosan beads (MICB) for arsenic removal. The maximum adsorption capacity of the MICB was calculated to be 35.7 mg g^−1^ for As(v), and 35.3 mg g^−1^ for As(iii) at pH 6.8. When the groundwater was used to examine the arsenic removal ability of MICB, the initial arsenic concentration of 0.103 mg L^−1^ in water could be reduced to less than 0.01 mg L^−1^ after 5 h with an adsorbent dosage of 1 g L^−1^.^150^

Cellulose is regarded as one of the most affordable raw materials available for the preparation of various functional materials.^151^ The abundant hydroxyl groups on the cellulose can be used directly or modified with other functional groups to extract toxic metal ions from water. In recent years, there is a growing interest in the utilization of natural lignocellulose materials as cheap and environment-friendly adsorbents.^152^ Agricultural residue materials such as straws, corn stalks, sugarcane bagasse and sawdust are abundant and readily available as natural resources for potential applications.^153^ For most of the natural cellulose materials, pretreatment by NaOH solution is a good way of increasing the specific surface area and to make the hydroxyl group more easily accessible for modification.^154^ The wheat straw was used to prepare a magnetic adsorbent with different Fe_3_O_4_ contents on the surface and used for arsenic extraction.^155^ Interestingly, the authors observed that Fe_3_O_4_ loading onto the wheat straw exhibited much higher adsorption capacity of As(v) (24–30 mg As/g Fe_3_O_4_) than the bare Fe_3_O_4_ (6–7 mg As/g Fe_3_O_4_). A novel Fe_2_O_3_ impregnated cross-linked cellulose was prepared by precipitation method for As(v) removal from water.^156^ The results indicated that Temkin model agreed for the adsorbate–adsorbent system, revealing the process of adsorption is a physicochemical process involving the hydroxyl (–OH) groups of the adsorbent surface. A clear relationship was found between the surface acidic groups and iron content. After modifying the jute fibre surface with succinic anhydride to incorporate carboxyl groups, the maximum iron loading was increased from 102 mg g^−1^ to 208 mg g^−1^.^157^ A list of iron modified biocomposite materials for arsenic adsorption is shown in Table 5.

**Table 5:** List of iron modified biocomposite materials prepared and used for arsenic adsorption

Adsorbents	pH	Initial conc. of As (mg L^−1^)	Iron loading content (mass, %)	Capacity (mg g^−1^)		Ref.
				As(iii)	As(v)	
Iron modified jute fibre	7.0	100	18.1	12.7		156
Fe_3_O_4_ coated wheat straw	6–8	As(iii): 28	—	3.9	8.1	157
		As(v): 28				
Nano-iron/oyster shell	6.8	As(iii): 1.8	50.2	0.9	—	158
Iron oxide coated fungal biomass	7.2	As(iii): 1.3	—	5.4	10.3	159
		As(v): 0.9				
ZVI nanoparticles modified starch	5.0	As(iii): 2	—	12.2	14	160
		As(v): 2				
Iron-loaded orange peel	3.0, 10.0	—	5.6	68.2	68.6	161
FeCl_3_ treated chestnut shell	9.0	As(iii): 100	—	0.9	—	162

#### 2.4.4. Iron oxy-hydroxides doped polymers adsorbents

Macroporous copolymers can be used as an adequate host material for the production of spherical beads of different geometry, design of textural properties and possibility of reaction with other functional groups.^163^ Taleb *et al.* (2015)^164^ synthesized anhydrous iron oxide impregnated poly-glycidylmethacrylate cross-linked resin, which was prepared by the radical suspension copolymerization, and reacted with diethylenetriamine (DETA) in tetrahydrofuran (THF) solvent. After the drop-wise addition of Fe(ii) solutions, NaHCO_3_ buffer solution was added to precipitate iron oxy-hydroxide in goethite form. The newly synthesized composite has the BET surface area of 178 m^2^ g^−1^. The pH_pzc_ was observed to decrease after arsenic adsorption, indicating a specific adsorption mechanism.^164^

**Fig. 5:**
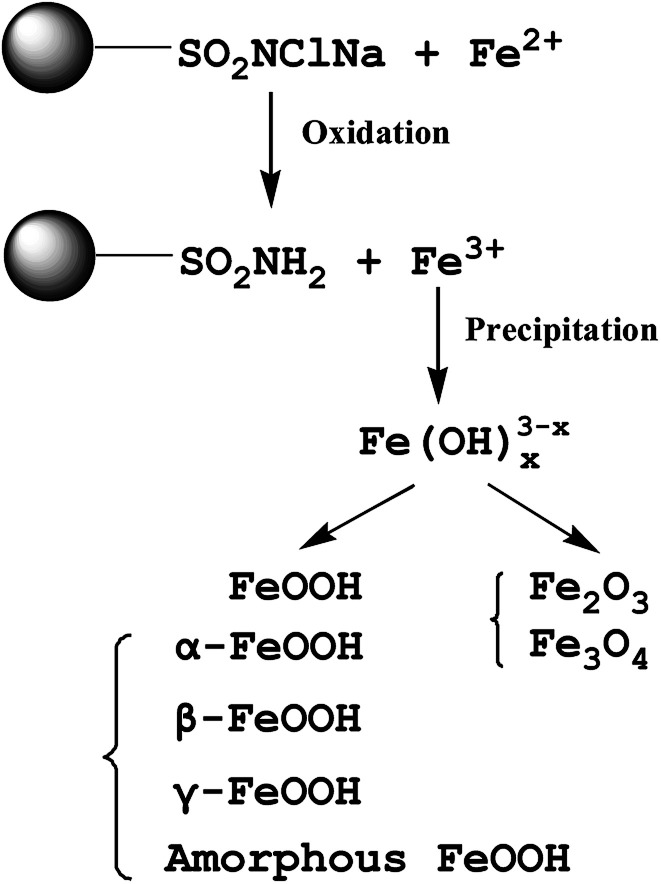
The schematic diagram of iron oxides deposited on poly(styrene-divinylbenzene) (St/DVB) matrix.

Recent studies on arsenic removal using anion exchange resins and fibres showed interesting results.^165,166^ Ociński *et al.* (2014)^167^ synthesized a hybrid polymer by dispersing iron oxides into a poly(styrene-divinylbenzene) (St/DVB) matrix. The supporting polymer contained sulfonamide groups (–SO_2_NH_2_ 2.3 mmol g^−1^), and sulfonic acid groups (–SO_3_^−^ 0.3 mmol g^−1^), which led to the incorporation of 12% Fe content in the matrix. The maximum adsorption capacity for this adsorbent calculated by Sips model^168^ reached 26.14 and 10.88 mg g^−1^ for As(iii) and As(v), respectively. The presence of interfering ions such as chlorides, sulfates, bicarbonates and carbonate ions did not show any influence on both As(iii) and As(v) adsorption, but a low concentration of phosphate anions caused an essential drop in As(v) removal efficiency. A schematic diagram of the simple two-stage synthetic process is shown in Fig. 5.

**Fig. 6:**
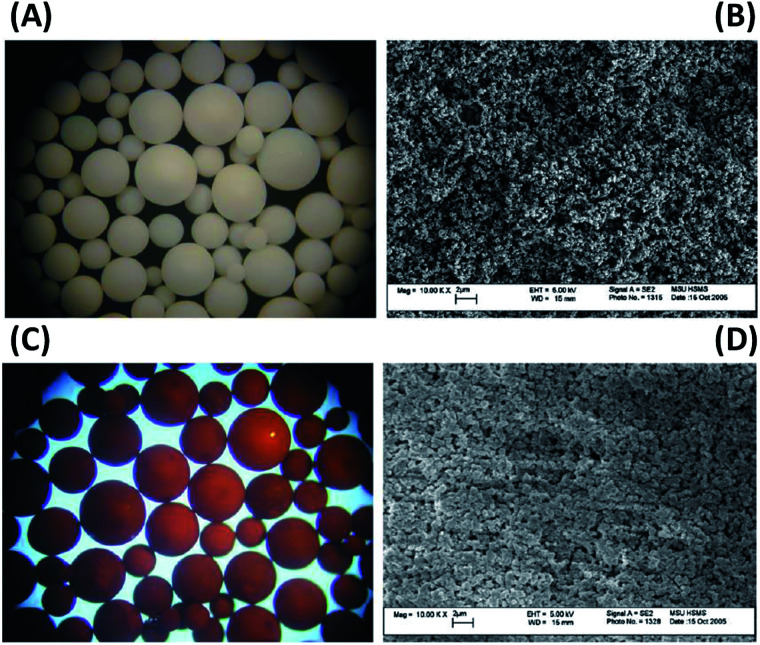
(A) Light microscope photograph of SBA (45×); (B) SEM images of SBA and (C) light microscope photograph of HFO/SBA adsorbent (45×); (D) SEM images of HFO/SBA adsorbent.^169^

The arsenic removal capacity was not always proportional to iron loading content. For example, Hu *et al.* (2017)^169^ prepared hydrated ferric oxide (HFO) loaded polymer, and observed that the adsorption capacity of As(v) increased with an increase of Fe mass percentage from 3 to 15%, but a further increase of Fe content resulted in a significant decline of the adsorption capacity. Similar results were also found using a HFO loaded strong base anion (SBA) exchange resin with a total Fe content of 318 mg Fe/g dry adsorbent.^170^ The comparison of SBA support and HFO/SBA adsorbent by microscopy was shown in Fig. 6, the HFO/SBA adsorbent developed a deep brown colour due to the dispersion of HFO particles. In order to overcome the drawback of nanoparticles used alone, superparamagnetic Fe_2_O_3_ nanoparticles dispersed cellulosic sponges were prepared and tested.^171^ The adsorption capacities of cellulose–Fe_2_O_3_ adsorbent were 2.11 mmol g^−1^ and 12.09 mmol g^−1^ for As(iii) and As(v), respectively, which are higher than that of iron nanoparticles in suspension.^171^

Kumar *et al.* (2016)^172^ reported an iron–aluminium hydroxides coated macroporous polyacrylamide for arsenic adsorption. The *in situ* chemical co-precipitation method was used for preparing iron–aluminium hydroxides particles by adding 25% NH_4_OH to iron and aluminium salts solution. The synthesized adsorbent showed an experimental maximum adsorption capacity of 82.3 and 49.6 mg g^−1^ for As(iii) and As(v), respectively. Anirudhan *et al.* (2013)^173^ observed that the incorporation of Fe(iii) ions could enhance the porous structure and increase the specific area of Fe(iii)-coordinated cellulose adsorbent from 21.7 to 31.6 m^2^ g^−1^. The maximum adsorption capacity for As(v) calculated by Langmuir isotherm equation was 105.47 mg g^−1^. The synthesized adsorbent has been tested using a simulated groundwater sample with no significant decrease in adsorption capacity.

#### 2.4.5. Iron oxy-hydroxide doped mineral oxides adsorbents

Mineral materials such as sand, rock, and clay materials have been used in water purification because of their low cost and high abundance in Nature.^174^ However, these materials exhibit low adsorption efficiency for arsenic because of the negative surface charge.^175^ Thus, application of composite adsorbents doped with iron ions was investigated in recent years.^176,177^ Fe-polycations modified montmorillonite adsorbent was synthesized by dispersing montmorillonite in the Fe-polycation solution and the maximum adsorption capacity of the composite was found to be 16.1, 15.3 mg g^−1^ for As(iii) and As(v), respectively, within the pH range of 4–10.^178^ Iron oxide coated natural rock (IOCNR) was synthesized through hydrothermal method.^179^ When the column was treated with an initial As(iii) concentration of 0.6 mg L^−1^, an up-flow rate of 8 mL min^−1^, and a bed depth of 20 cm, the breakthrough point (0.01 mg L^−1^) occurred after 63 h and the exhausted point (90% of the initial concentration, *i.e.* 0.54 mg L^−1^) occurred after 110 h, indicating IOCNR is suitable for arsenic removal from water. A modified iron-coated sand (DMICS) was also synthesized by dynamic soaking of iron onto the sand,^180^ Temkin isotherms were used to describe the equilibrium studies better than Langmuir and Freundlich models, the maximum adsorption capacity was calculated to be 5.6 mg g^−1^. An iron hydroxide modified diatomite was prepared with the iron loading amount of 10% and 17%.^181^ A maximum capacity of 40.82 mg g^−1^ was obtained at pH 4 and 17% iron loading amount.

An *in situ* remediation for arsenic from groundwater by using an aquifer iron coating method was considered as an effective and simple way for arsenic remediation in rural and remote areas where groundwater is used as the main water resource for drinking.^182^ A continuous injection of FeSO_4_ and NaClO solutions for 96 h led to the formation of a uniform α-FeOOH (30–50 nm) coating on the surface of the sand. During this process, ferrous iron can also be adsorbed and subsequently oxidized to form new ferric hydroxide particles, which can be used to adsorb arsenic from water. The process of adsorption/co-precipitation with fine goethite particles resulted in arsenic immobilization.^182^ Titanium dioxide (TiO_2_) is a famous photocatalyst that offers a relatively inexpensive and environmentally safe way to achieve oxidation of As(iii) to As(v).^183^ TiO_2_ nanoparticles doped with 10% Fe adsorbent could effectively oxidize As(iii) to As(v).^184^ The maximal adsorption capacities calculated by Langmuir isotherm model were 8.61 and 17.35 mg g^−1^ for As(iii) and As(v), respectively. The presence of SO_4_^2−^ anion hindered the adsorption of only As(iii), while PO_4_^3−^ anion decreased the adsorption capacities of both As(iii) and As(v) species from water.^184^

In the case of Fe doped materials, X-ray mapping, EDX and XPS methods are frequently used to determine the surface concentration of Fe ions. For example, Fan *et al.* (2018)^185^ used X-ray mapping to investigate the distribution of Fe in the carbon matrix. Gallios *et al.* (2017)^186^ employed X-ray mapping to demonstrate that the impregnated iron was uniformly distributed on the internal surface of the granular activated carbon. Li *et al.* (2013)^187^ used XPS to create the elemental map of magnetic nanoparticles impregnated with N-doped porous carbon.

## 3. Adsorption mechanisms

Many studies demonstrated that arsenic adsorption on iron-based materials occurs through formation of inner-sphere complexes such as monodentate, bidentate, or tridentate bonds.^188,189^ X-ray absorption spectroscopy (XAS) and X-ray photoelectron spectroscopy (XPS) techniques are commonly used to investigate the mechanism including the formation of different types of complexes and the redox transformation of adsorbed As on adsorbents.

**Fig. 7:**
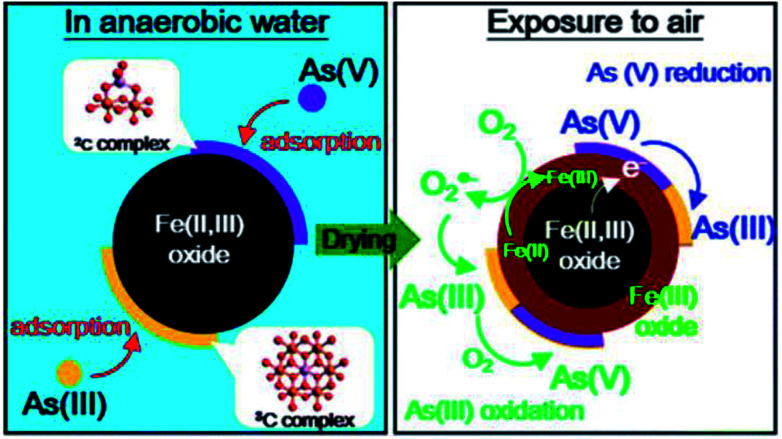
Mechanism of arsenic adsorption on magnetite nanoparticles in anaerobic water and air-enriched water.^190^

Liu *et al.* (2015)^190^ demonstrated that arsenic adsorption on magnetite nanoparticles (MNPs) is an endothermic process. The X-ray absorption fine structure (EXAFS) spectra suggested that As(v) adsorption on MNPs mainly through the formation of bidentate binuclear corner-sharing complexes (^2^C) with the typical interatomic Fe–As distance of 3.35–3.39 Å. As(iii) adsorption on MNPs occurs through tridentate hexanuclear corner-sharing (^3^C) complexes with the typical inter-atomic Fe–As distance of 3.49–3.67 Å. The typical inter-atomic Fe–As distance of the bidentate binuclear corner-sharing complexes (^2^C) is ∼3.3–3.4 Å, and that of the monodentate mononuclear corner-sharing complexes (^1^V) is ∼3.5–3.6 Å.^190^ For the arsenic loaded MNPs exposed to air, XANES and XPS results revealed the complex redox transformation of the adsorbed arsenic, which was shown in Fig. 7. XPS spectra were used to investigate the mechanism of As(v) adsorption on MNPs.^191^ It was revealed that the surface oxygen and iron atoms act as Lewis acids, while arsenate anions, act as a Lewis base. The specific adsorption reaction was formed through the formation of inner-sphere complexes. The monoprotonated bidentate complexes were dominant and no reduction of As(v) was observed on the surface of the MNPs. Extended X-ray Absorption Fine Structure (EXAFS) spectra suggested the predominant formation of bidentate binuclear corner-sharing complexes (^2^C) for As(v), and tridentate hexanuclear corner-sharing (^3^C) complexes for As(iii) on MNP surfaces.^191^ Also, As(v) can be reduced to As(iii) because of the role played by the reactive Fe(ii). Thi *et al.* (2015)^192^ compared the adsorptive performance of Fe_3_O_4_ and Mn, Cu doped Fe_3_O_4_ nanoparticles for As(iii) wastewater treatment. The paper reported that Cu doped Fe_3_O_4_ nanoparticles have higher adsorption capacity towards arsenic than Fe_3_O_4_ and Mn-doped Fe_3_O_4_ nanoparticles because the substitution of Cu^2+^ ions for smaller radii of Fe^2+^ could increase the porosity and specific surface area of Cu doped Fe_3_O_4_ nanoparticles. The saturation magnetic moments of the adsorbent decreased from 65.9 emu g^−1^ to 53.2 emu g^−1^ after doping with Cu ions.^192^

The arsenic adsorption on synthetic siderite was greatly enhanced from ∼10 mg g^−1^ to around 120 mg g^−1^ under oxidizing conditions, while 75% of the siderite was transformed to goethite during arsenic adsorption.^193^ The EXAFS spectra indicated the bidentate binuclear corner-sharing complexes (^2^C) and mononuclear corner-sharing complexes (^1^V) are involved during As(iii)/As(v) adsorption on siderite. In aerobic conditions, Fe(ii) was gradually oxidized to Fe(iii), the As(iii) oxidation and complexes between Fe(ii)–Fe(iii) minerals greatly improved arsenic adsorption.^193^ As(v) adsorption on hematite and goethite through the mechanism of ligand exchange.^194^

Besides of surface complexation in arsenic adsorption, electrostatic attraction and ion exchange are also contributed to the arsenic removal. The isoelectric point (pH_iso_) is an important factor influencing the adsorption capacity and rates. For example, Fe_2_O_3_ crystalline structures exhibited pH_iso_ of around 7.2, while the synthetically derived Fe_2_O_3_ typically exhibits an pH_iso_ between 8.1 and 8.8.^195^ The pH_iso_ of typical iron (oxy)hydroxide such as goethite (α-FeOOH) and magnetite was reported to be 6.9 and 6.4, respectively.^195^ β-FeOOH, which was demonstrated an excellent binding capacity for As(iii) and As(v), the pH_iso_ was determined to be 8–9. It is understandable that adsorbents with high isoelectric points could decrease the electrostatic repulsion forces between the adsorbent surface and negatively charged arsenic species in pH environments greater than pH_iso_.^196^

Ion exchange is also an important mechanism for arsenic adsorption,^197^ especially of the iron-based layered double hydroxides (LDHs) which are theoretically the best anion exchangers due to their potential to host arsenic anions in their interlayer space, which considerably increase their anion removal performance. This ability of the interlayer space to host arsenic anions makes LDHs superior to a majority of anion exchangers.^197^ The schematic mechanism of H_2_AsO_4_^−^ adsorption on typical Mg–Fe-LDHs was shown in Fig. 9. Zhu *et al.* (2015)^198^ prepared an iron-manganese binary oxide (FeMnOx) for arsenic adsorption with adsorption capacities of 47.05 and 49 mg g^−1^ for As(iii) and As(v) ions, respectively. The XPS spectra indicated that a portion of As(iii) was converted to As(v) in presence of MnO_2_. Wang *et al.* (2014)^199^ reviewed the mechanism of arsenic on Fe–Al binary metal oxides. The authors indicated that the presence of Fe^2+^ ions in FeO could reduce As(v) to As(iii) while Fe^2+^ is oxidized to Fe^3+^ in the form of Fe_2_O_3_ (eqn (9)).

9 Fe^2+^ + H_3_AsO_4_ → Fe^3+^ + H_3_AsO_3_ + OH^−^

**Fig. 8:**
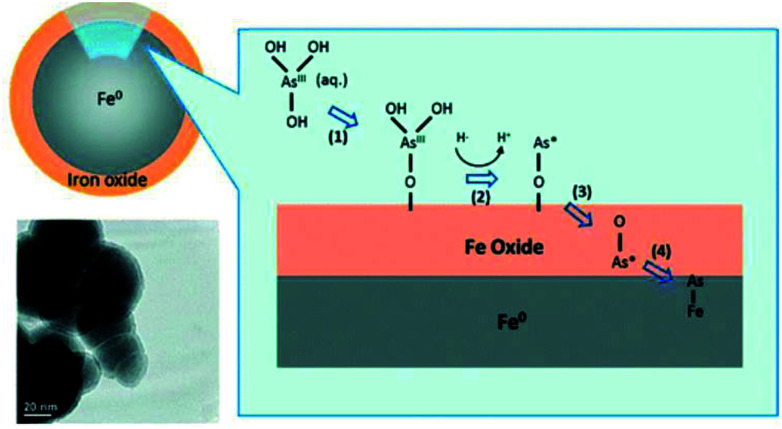
The schematic diagram of the adsorption of As(iii) species on nZVI particles.^200^

Yan *et al.* (2012)^200^ investigated the mechanism of As(iii) adsorption on nZVI nanoparticles using XAS method, the authors revealed that As(iii) species underwent two stages of transformations after As adsorption on the nZVI surface. The As–O bonds are broken and the arsenic species are further reduced and diffused across the thin iron oxide layer, which resulted in the formation of As–Fe bonds. Different arsenic valence states of As(0), As(iii), and As(v) were observed in nZVI after As(iii) adsorption. As(iii) was distributed throughout the oxide shell, As(0) was embedded at the interface of Fe(0) core and iron oxide shell, and As(v) existed primarily in the iron oxide layer.^200^ The mechanism of As(iii) adsorption on nZVI nanoparticles was shown in Fig. 8. The inner-sphere complexation between arsenic and iron compounds was demonstrated by many researchers and summarized in Table 6.

**Fig. 9:**
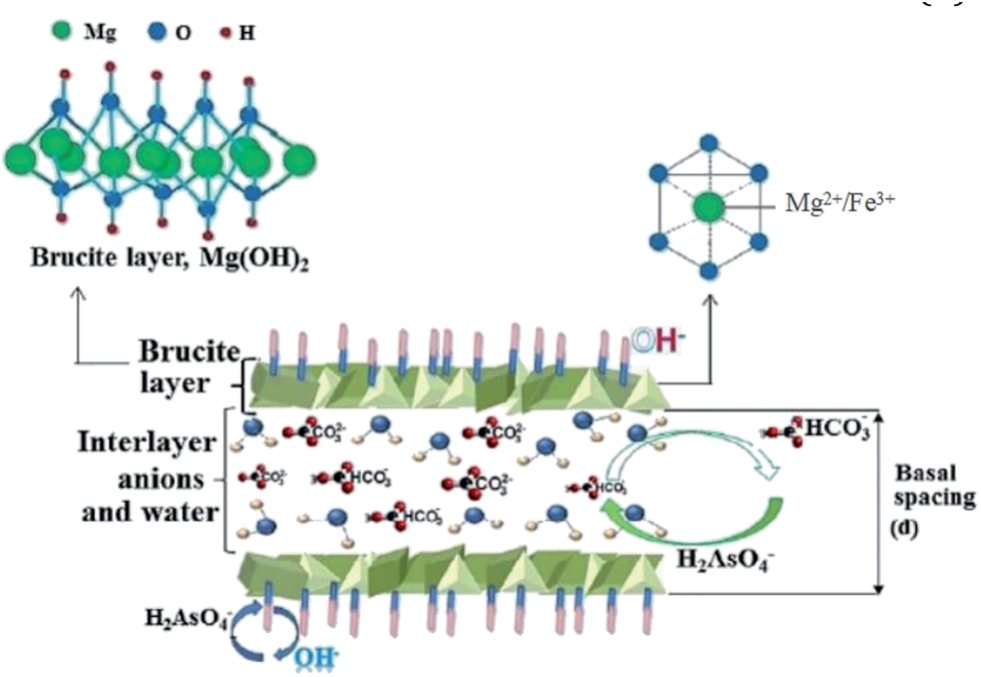
Schematic mechanism of As(v) adsorption on Mg–Fe–CO_3_^2−^-LDHs.^197^

**Table 6:** Inner-sphere complexation constants for arsenic adsorption on iron oxide minerals^201–204^

Adsorption reaction	Complexation constants			
	α-FeOOH	Fe_3_O_4_	Fe(OH)_3_	HFO
≡FeOH + H^+^ → ≡FeOH^+^_2_	7.47	4.60	—	7.29
≡FeOH → ≡FeO^−^ + H^+^	−9.51	−8.20	—	−8.93

**As(** **iii** **) adsorption constants**				
≡Fe–OH + H_3_AsO_3_ → ≡Fe − H_2_AsO_3_ + H_2_O	39.93	38.41	40.20	38.76
≡Fe–OH + H_2_AsO_3_^−^ → ≡Fe − HAsO_3_^−^ + H_2_O	32.4	33.02	—	31.87

**As(** **v** **) adsorption constants**				
≡Fe–OH + H_3_AsO_4_ → ≡Fe − H_2_AsO_4_ + H_2_O	31.00		29.31	29.88
≡Fe–OH + H_2_AsO_4_^−^ → ≡Fe − HAsO_4_^−^ + H_2_O	26.81		23.51	24.43
≡Fe–OH + HAsO^2^_4_^−^ → ≡Fe − AsO^3^_4_^−^ + H_2_O	20.22		10.58	18.10

## 4. Separation of iron-based magnetic adsorbents from water

Separation of the pollutant-laden adsorbents from water was always considered as a major challenge for drinking water treatment processes.^205^ It is important to separate the pollutant saturated adsorbents out of reactor and distribution systems to avoid causing the secondary environmental pollutions through the disposal of these materials.

Magnetite nanoparticles (MNPs) are promising adsorbents for As removal because of their high adsorption capacity and easy separation from water under of a low external magnetic field.^206^ Sepúlveda *et al.* (2018)^207^ compared the magnetic property of Fe_3_O_4_ and Cu doped Fe_3_O_4_ nanoparticles. The saturation magnetic moments of the adsorbent decreased from 65.9 emu g^−1^ to 53.2 emu g^−1^ after doping with Cu ions. Magnetic nanocomposite (MNC) was synthesized by modifying Fe_3_O_4_ with 3-amino propyltrimethoxysilane for toxic metals removal from water.^208^ A hybrid Fe_3_O_4_–chitosan adsorbent showed higher affinity toward As(iii) compared to As(v), and the adsorption behaviour was well described using both Langmuir and Freundlich isotherms.^209^ γ-Fe_2_O_3_ embedded biochar could be easily separated from the solution by a magnet. The saturation magnetization of the γ-Fe_2_O_3_/biochar composite was determined to be 69.2 emu g^−1^, which was very close to that of pure γ-Fe_2_O_3_ materials (76.0 emu g^−1^).^210^ A Fe_3_O_4_ loaded wheat straw showed typically superparamagnetic behaviour with the loop area being zero.^211^ The saturated magnetizations reached 6.18, 9.12 and 11.87 emu g^−1^, respectively, depending on the Fe_3_O_4_ content.

## 5. Future research needs on technology development

Development of simple and inexpensive water treatment systems is very important for providing potable water to millions of people. So far, most research efforts were focused on developing novel adsorbent materials with high adsorption capacities. Fewer efforts have focused on regeneration or disposal of arsenic-bearing sludge. Among the mentioned iron-based adsorbents, iron oxide, the iron doped activated carbon, and iron modified chelating resin were most widely used in practice for arsenic removal from drinking water. Two commercial inorganic adsorbents – Activated Alumina (AA) and Granular Ferric Hydroxide (GFH) are most widely used for arsenic adsorption. These materials currently allow the maximum allowable concentration (MAC) of arsenic (10 μg L^−1^) to be achieved. The pilot system showed adsorption based on GFH exhibited good performance in removing arsenic from groundwater. The initial concentration of 400 μg L^−1^ was reduced to less than 20 μg L^−1^ in most of the tube wells.^212^ Natural hematite, magnetite, and goethite, which are suitable for both As(iii) and As(v) removal, were also expected to be used in practice due to abundant presence. The major disadvantages are the comparatively low adsorption capacity and very long time requirement (∼2 days) to reach the equilibrium.^213^ Iron(iii)-loaded chelating resin were used for As(iii) removal from drinking water, and up to 98% removal of As(iii) was achieved at pH 6.0.^214^ Notably, the range of costs for these adsorbents varied widely while few adsorbents can be produced at the relatively lower costs. Therefore, the “low-cost” and “easy-to-use” technologies for arsenic removal in the low- and lower medium-income countries are very important for future development of iron-based adsorbents.

Moreover, use of nanomaterials for arsenic adsorption has been explored in recent years, but the nanostructured adsorbents tend to agglomerate together, which decrease the adsorption and removal efficiency. Therefore, loading nanoparticles onto appropriate supporting materials is becoming a feasible strategy with the advantages of high reactivity and easy separation from water. The research has to continue for developing such adsorbents based technologies to be applied in the field in a sustainable manner.

## 6. Conclusions

A brief review of the removal of arsenic ions from water using iron-based adsorbents has been presented. A few adsorbents discussed in this review include iron compounds such as iron oxides, oxy-hydroxides such as amorphous hydrous ferric oxide (FeOOH), goethite (α-FeOOH), hematite (α-Fe_2_O_3_), iron-based LDHs, zero-valent iron nanoparticles, iron-doped activated carbon, biocomposite materials, iron-doped polymers and iron-doped mineral oxides. Relative advantages and disadvantages of the iron-based adsorbents used for the removal of arsenic from water have been mentioned. The mechanism of arsenic adsorption on iron-containing adsorbents was summarized as the formation of inner-sphere complexes such as monodentate, bidentate and tridentate complexes. Roles of Fe(0), Fe(ii) and Fe(iii) in the oxidation of As(iii) to As(v) and its extraction efficiency are also included.

Iron-oxyhydroxide doped biosorbents yielded interesting results, considering their abundance and low cost. For the iron modified adsorbents, a thorough investigation on leaching of ions from the adsorbents has to be conducted to understand the stability of the adsorbent under different experimental conditions. Iron compounds and iron-based LDHs have higher arsenic adsorption efficiencies, but they are usually difficult to remove from water owing to the nano- to micrometer size of the particles. Overall, there exist significant progress and benefit on using iron loaded biomass or polymers for removing arsenic species from groundwater in a practical way to make potable water accessible for the rural population.

## Conflicts of interest

There are no conflicts to declare.

## Supplementary Material
